# Supplementary material for: The β2-Subunit (AMOG) of Human Na+, K+-ATPase Is a Homophilic Adhesion Molecule
Source: Int J Mol Sci. 2022 Jul 14;23(14):7753. doi: 10.3390/ijms23147753 (PMC9322774; doi:10.3390/ijms23147753)
Supplement: Supplementary file 1 [file ijms-23-07753-s001.zip › ijms-1772234-supplementary.pdf]

The β2-subunit (AMOG) of human Na^+,K^+-ATPase is a homophilic adhesion molecule

María Luisa Roldán[1], Gema Lizbeth Ramírez-Salinas[2], Marlet Martinez-Archundia[3], Francisco Cuellar-Perez[1], Claudia Andrea Vilchis-Nestor[1], Juan Carlos Cancino-Diaz[4] and Liora Shoshani[1*].

1 Department of Physiology, Biophysics and Neurosciences, CINVESTAV-IPN, 2508 IPN Ave., San Pedro Zacatenco, México city, 07360, DF, Mexico. mroldan@fisio.cinvestav.mx, pacupe_1@hotmail.com; cvilchis85@gmail.com 2 Department of Immunology, Instituto de Investigaciones Biomédicas, Universidad Nacional Autónoma de México (UNAM). gemali86@hotmail.com 3 Laboratorio de Modelado Molecular, Bioinformática y diseño de fármacos. Departamento de Posgrado Escuela Superior de Medicina del Instituto Politécnico Nacional. mtmartineza@ipn.mx 4 Departamento de microbiología, Escuela Nacional de Ciencias Biológicas del Instituto Politécnico Nacional. jcancinod@ipn.mx

* Correspondence: shoshani@fisio.cinvestav.mx; Tel.: (+52 55 5747 3360)

Supplemental Information

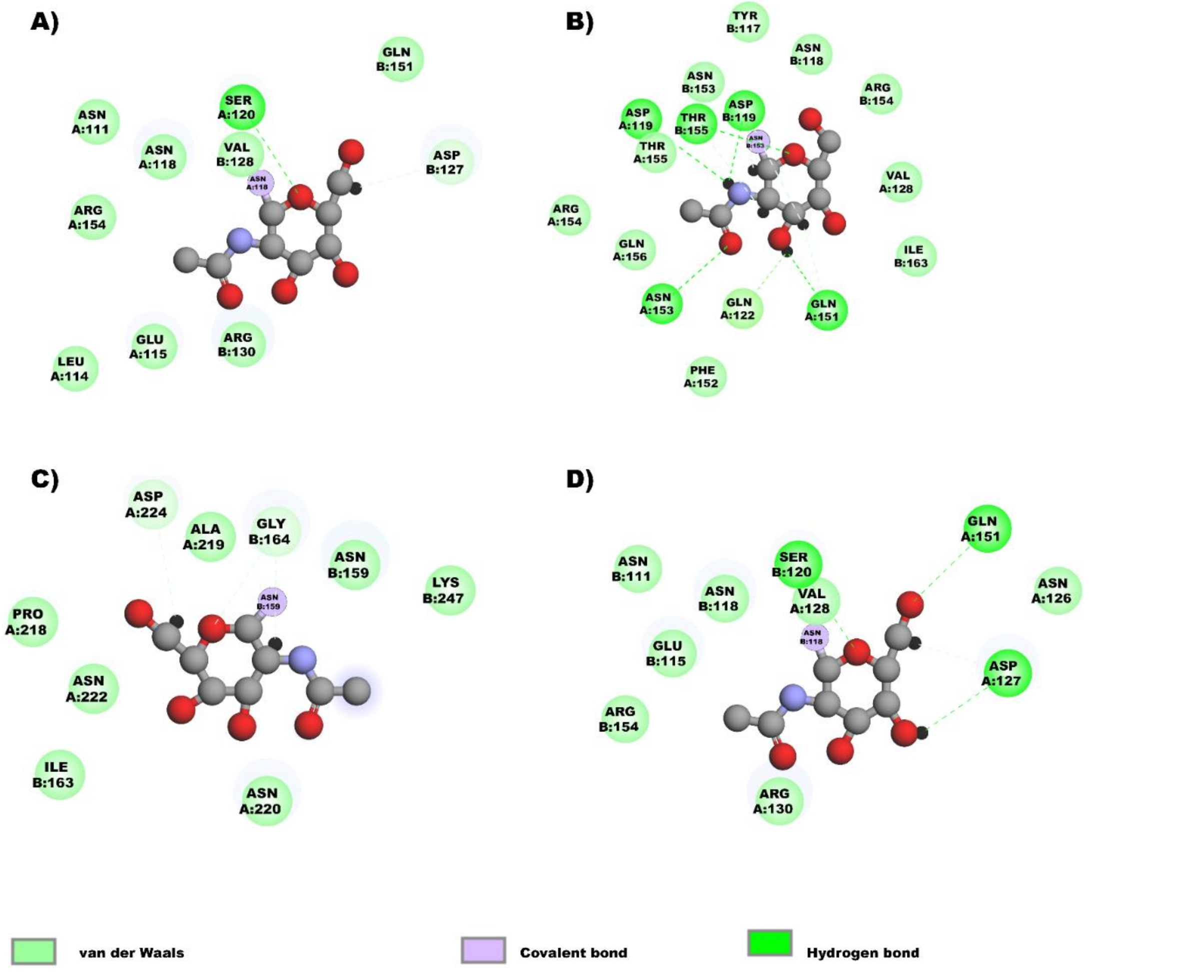

Figure S1: Glycosylation sites at the interface of EDβ2-EDβ2 trans-dimer. A) Glycosylated Asn118 (Chain A) shows the following interactions: hydrogen bonds with Ser120(A), Van der Waals interactions with Chain B (Val128, Gln151, Arg130, and Asp127). B) Glycosylated Asn153 (Chain B) shows hydrogen bond interaction with Asn153 (Chain A), and the following Van der Waals interactions with (Val128A, Gln151A, Gln122A, Phe152A, and Gln156A). C) Glycosylated Asn159 (Chain B) forms Van der Waals interactions with Asn222A, Pro218A, Asn220A, Ala219A, and Asp224A. D) Glycosylated Asn118 (Chain B) shows mainly intramolecular interactions and forms hydrogen bonds with Ser120B, Gln151A, and Asp127A and some weaker interactions with Asn126A and Arg130A. Discovery Studio [46)].
